# Richard Harrison

**Published:** 1825-02

**Authors:** 


### OBITUARY.

DIED lately, at his house in Argyll-street, RICHARD HARRISON, M.D. F.L.S. Fellow of the Royal College of Physicians. He had long laboured under a pulmonary complaint, and died rather suddenly from hemorrhage from the lungs.

Dr. Harrison was educated under the direction of his uncle, receiving the first principles of his profession at the Horncastle Dispensary; thence he was sent to Oxford, where he remained till he took out his degree. He spent several sessions in attending the lectures in Edinburgh; and afterwards went abroad for some years. Notwithstanding the precarious state of his health, he was enthusiastically attached to his profession, and devoted himself to its cultivation with unremitting industry; having been found by the writer of this notice, on the day he died, occupied with his usual studies, although confined to bed, and in a state of extreme debility. He has left a considerable number of manuscripts, and had been occupied for some time in preparing a work for the press on the subject of Hydrocephalus, but which, we believe, is not in a state of sufficient forwardness for publication.

